# Complete genome sequence of *Limnobacter thiooxidans* CS-K2^T^, isolated from freshwater lake sediments in Bavaria, Germany

**DOI:** 10.1128/mra.00992-23

**Published:** 2023-12-04

**Authors:** Miu Naruki, Aoi Watanabe, Tomoro Warashina, Teppei Morita, Kazuharu Arakawa

**Affiliations:** 1 Institute for Advanced Biosciences, Keio University, Tsuruoka, Yamagata, Japan; 2 Systems Biology Program, Graduate School of Media and Governance, Keio University, Fujisawa, Kanagawa, Japan; 3 Faculty of Environment and Information Studies, Keio University, Fujisawa, Kanagawa, Japan; University of Delaware College of Engineering, Newark, Delaware, USA

**Keywords:** *Limnobacter thiooxidans*, complete genome, nanopore sequencing, bacterial genome, thiosulfate oxidation

## Abstract

*Limnobacter thiooxidans* CS-K2^T^ is a Gram-negative bacterium first isolated from the sediment of the littoral zone of a freshwater lake in Germany. We here present the complete annotated genome sequence of this thiosulfate-oxidizing bacterium, spanning 3.54 Mb and encoding 3,192 protein-coding sequences.

## ANNOUNCEMENT

*Limnobacter thiooxidans* CS-K2^T^ DSM 13,612 is a gram-negative, thiosulfate-oxidizing bacterium with slightly curved rod shape and motile by a single polar flagellum. This Burkholderiaceae bacterium was first isolated from sediment samples in the littoral zone of a freshwater lake, Lake Chimessee, in Bavaria, Germany (1).

In this study, strain CS-K2^T^ was obtained from DSMZ German Collection of Microorganisms and cell cultures (GmbH). A single colony was extracted and grown over 52 hours at 30°C in a Marine Broth 2216 medium (Millipore) supplemented with 0.2% succinic acid, as the bacteria cannot be cultured under the presence of glucose. A culture of an optical density of 1.446 (18 mL) were collected, and genomic DNA was purified using Genomic-tip 20/G (QIAGEN, 10223) according to the manufacturers protocol for both of the long- and short-read sequencing. Long-read sequencing library was prepared using Rapid Barcoding Kit (SQK-RBK004; Oxford Nanopore Technologies) and sequenced with GridION device using R10.3 Flowcell (FLO-MIN111). Subsequently, the reads were basecalled, demultiplexed, and trimmed for the adaptor sequences using Guppy v.6.3.8. Illumina sequencing was performed for error correction using the HyperPlus library preparation kit (Kapa Biosystems) and a MiSeq sequencer with Reagent Kit v3 600 cycles as 300 bp paired-ends (Illumina). The quality of the long reads was assessed using Nanoplot version 1.20 (2), resulting in a total of 924,125,614 bp consisting of 129,906 reads of N50 length of 11,878 bp. Reads longer than 10,000 bp with an estimated coverage of 264× were used for assembly using Canu version 2.2 (3). All raw Illumina short reads (9.2M reads) were used to polish the assembly with Burrows-Wheeler Aligner version 0.7.11 (4), indexed with SAMtools version 1.11 (5), and followed by two rounds of polishing with Pilon version 1.23 (6). The completeness of the assembly was assessed with CheckM version 1.0.18 and DDBJ Fast Annotation and Submission Tool (DFAST) pipeline was utilized to conduct genome annotation on functional sequences (7, 8). Completeness was 99.4% with Betaprotobacteria marker lineage. All software were used with their default settings unless otherwise specified.

The assembled genome had a total length of 3,536,915 bp and a G + C content of 53.2%, containing 3,192 protein-coding sequences, six rRNAs, and 41 tRNAs. Interestingly, the ANI between *L. thiooxidans* and other species from the same genus was found to be relatively low, at 81.2% (*Limnobacter alexandrii*) and 78% (*Limnobacter humi*), indicating that despite their shared genus, they are not highly similar. According to the DFAST annotations, we identified genes such *soxY*/*soxZ* and *soxB* which are thiosulfate oxidation carrier proteins and thiosulfohydrolase, respectively (9, 10). They play a vital role in the thiosulfate oxidation pathway as thiosulfate is involved with the sulfur cycle in marine sediments as it can be converted to sulfate by microorganisms to generate energy (11).

## Data Availability

The genome sequences reported here were deposited in DDBJ under accession number AP028947, and the raw reads were deposited in the Sequence Read Archive (SRA) under BioProject accession number PRJNA1013011 as SRR25907068 and SRR25907069 runs.
